# P047. Paroxysmal episodic hemicrania in a child. A complex differential diagnosis

**DOI:** 10.1186/1129-2377-16-S1-A100

**Published:** 2015-09-28

**Authors:** Roberto Frusciante, Alessandro Capuano, Catello Vollono, Federico Vigevano, Samuela Tarantino, Massimiliano Valeriani

**Affiliations:** Headache Centre, Bambino Gesù Children's Hospital, Rome, Italy; Department of Geriatrics, Neurosciences & Orthopedics, Unit of Neurophysiopathology and Sleep Medicine, Catholic University, Policlinico Agostino Gemelli, Rome, Italy; Centre for Sensory-Motor Interaction, Aalborg University, Aalborg, Denmark

## Background

Headache is a common disease in children. Differential diagnosis of primary headaches in children is challenging due to some peculiar features of headache at that age. We describe a case in a child who presented with headache resembling characteristics of migraine without aura, paroxysmal hemicrania and cluster headache.

## Methods and results

An 11-year-old boy referred to our Headache Centre suffering from headache for the past 5 years. Headache characteristics were: frontal pain, constricting in quality and excruciating pain intensity, at times vomiting. Duration of attacks was referred ranging from 20 to 40 minutes, headache occurred daily during the last 2 months. Attacks recurred many times daily. Attacks of headache occurred in periods lasting from 1 to 2 months, separated by pain-free periods lasting 1 month. During the headache attacks, the child presented eyelid oedema and nasal congestion. Personal medical history was negative. Familial history was positive for migraine without aura (paternal aunt). General and neurological examination, including fundus oculi, were normal. MRI scan resulted normal. Previous prophylaxis with pizotifen, amitriptyline, verapamil, topiramate and prednisone were ineffective, instead indomethacin was effective.

## Discussion

In children, the characteristics of headache often overlap between different forms. Cluster headache is a rare form and very few cases in pediatric age have been described[1]. Our patient presented headache attacks with autonomic activation (eyelid oedema and nasal congestion), that usually rule out the diagnosis of migraine attacks. On the contrary, these features are thought to be specific of TACs. Furthermore, the clustering of attacks, more than one per day, the ineffectiveness of different prophylactic therapies, and the complete efficacy of therapeutic doses of indomethacin, support the diagnosis of paroxysmal episodic hemicrania in our patient.

Written informed consent to publication was obtained from the patient(s).
